# A scoring system to predict resistance to ceftolozane/tazobactam in respiratory isolates of *Pseudomonas aeruginosa*

**DOI:** 10.1093/jac/dkae476

**Published:** 2025-01-15

**Authors:** Eda Karadogan, Ahmet Sertcelik, Gulcin Telli Dizman, Hanife Uzar, Gulsen Hazirolan, Banu Cakir, Gokhan Metan

**Affiliations:** Department of Public Health, Division of Epidemiology, Hacettepe University Faculty of Medicine, Ankara, Turkey; Department of Public Health, Division of Epidemiology, Hacettepe University Faculty of Medicine, Ankara, Turkey; Department of Infectious Diseases and Clinical Microbiology, Hacettepe University Faculty of Medicine, Ankara, Turkey; Department of Public Health, Division of Epidemiology, Hacettepe University Faculty of Medicine, Ankara, Turkey; Department of Clinical Microbiology, Hacettepe University Faculty of Medicine, Ankara, Turkey; Department of Public Health, Division of Epidemiology, Hacettepe University Faculty of Medicine, Ankara, Turkey; Department of Infectious Diseases and Clinical Microbiology, Hacettepe University Faculty of Medicine, Ankara, Turkey

## Abstract

**Objectives:**

To develop a scoring system to predict resistance to ceftolozane/tazobactam in *Pseudomonas aeruginosa* strains isolated from respiratory specimens.

**Methods:**

A case–control study was conducted to evaluate the risk factors associated with resistance to ceftolozane/tazobactam. Patients with *P. aeruginosa* were defined as cases if they had ceftolozane/tazobactam-resistant strains, whereas those with ceftolozane/tazobactam-susceptible strains were defined as test-negative controls. A predictive scoring system based on binary logistic regression coefficients was formulated to predict resistance to ceftolozane/tazobactam. The score’s performance was assessed using ROC curves and AUC. The sensitivity, specificity and predictive values of the score were determined on the basis of a cut-off point, using the Youden index.

**Results:**

Ceftolozane/tazobactam resistance was detected in 18.4% of *P. aeruginosa* isolates from 473 patients. In multivariate analysis, a history of bronchoscopy [OR (95% CI) = 2.1 (1.1–4.3), *P* = 0.035], invasive mechanical ventilation [OR (95% CI) = 2.4 (1.2–4.5), *P* = 0.009], colistin/polymyxin B use [OR (95% CI) = 3.2 (1.8–5.7), *P* < 0.001] and fluoroquinolone use [OR (95% CI) = 2.3 (1.1–4.8), *P* = 0.024] in the preceding month prior to *P. aeruginosa* isolation were significantly associated with ceftolozane/tazobactam resistance. The AUC (95% CI) of the score was 0.734 (0.675–0.794), with a sensitivity of 69%, specificity of 71.8%, positive predictive value of 35.5% and negative predictive value of 91.1% at the cut-off point of 2, out of a range of 0–5.

**Conclusions:**

In respiratory tract infections caused by *P. aeruginosa*, use of the proposed scoring system may reduce inappropriate use of ceftolozane/tazobactam in empirical treatment.

## Introduction

Treatment of pneumonia caused by MDR *Pseudomonas aeruginosa* is a challenge.^1^ Ceftolozane/tazobactam is a treatment option for MDR *P. aeruginosa* infections in critically ill patients.^2,3^ In a randomized controlled trial, ceftolozane/tazobactam showed similar effectiveness to meropenem in the treatment of nosocomial pneumonia.^4^

Use of ceftolozane/tazobactam for *Pseudomonas* pneumonia may delay appropriate treatment when culture results are awaited. On the contrary, widespread use without susceptibility results may induce resistance. Ceftolozane/tazobactam resistance was found to be an independent risk factor for 90 day mortality in patients with MDR Gram-negative bacterial infections.^5^

Research on risk factors of ceftolozane/tazobactam resistance is still limited.^6,7^ In this study, we aimed to investigate the predictors of ceftolozane/tazobactam resistance in *P. aeruginosa* strains isolated from respiratory specimens to develop a clinical scoring system to use in empirical prescription.

## Materials and methods

### Study design and participants

This was a single-centre, case-test-negative control study. All patients aged 18 years and older, admitted to Hacettepe University Adult and Oncology Hospitals between 1 May 2019 and 31 May 2023, were included in the study population if *P. aeruginosa* positivity was reported in their sputum, deep tracheal aspirate (DTA) and/or bronchoalveolar lavage (BAL) fluid cultures. Those with ceftolozane/tazobactam resistance were identified as ‘cases’, whilst their counterparts with ceftolozane/tazobactam-sensitive strains were defined as test-negative controls.

### Setting

The study was conducted at Hacettepe University Adult and Oncology Hospitals, a tertiary care teaching centre located in Ankara, the capital of Türkiye, with a bed capacity of approximately 1200.

### Inclusion and exclusion criteria

Patients were included if they met the specified case or control definitions. Patients with missing clinical information and those with recurrent *P. aeruginosa* within 30 days were excluded from study. Four patients in the case group, and three in the control group had recurrent *P. aeruginosa* growth that was detected later than 30 days after the initial isolation; these seven isolates were included in the analyses as independent episodes, referred to as ‘patients’ in tables, for simplicity.

### Data collection, definitions and sampling

Patient data, including demographic and clinical characteristics, were recruited retrospectively from hospital electronic medical records. Factors associated with ceftolozane/tazobactam resistance were examined in the entire dataset, and further restricted data to monomicrobial isolates, i.e. *P. aeruginosa* was the only isolate.

Corticosteroid use was defined as the use of 20 mg or more of prednisone or its equivalent for more than 14 days. MDR was defined as resistance to at least one antimicrobial agent in at least three antimicrobial categories. XDR was defined as non-susceptibility to at least one agent in all but two or fewer antimicrobial categories (i.e. bacterial isolates remain susceptible to only one or two categories). PDR was defined as non-susceptibility to all agents in all antimicrobial categories.^8^

The percentages of antibiotic use in the last month were determined as 52% in the case group and 28% in the control group, based on literature.^7^ As there were no data in the literature on the use of any antibiotic in the last month between the ceftolozane/tazobactam-sensitive and -resistant groups, the use of third- and fourth-generation cephalosporins in the last 1 month in the reference study was used as a surrogate. With 95% confidence and 90% power, the minimum sample size was calculated as 72 for the case group and 144 for the control group. All eligible isolates were included in the analyses, resulting in a total of 87 cases and 386 controls.

### Species identification and antimicrobial susceptibility

All BAL, DTA and sputum specimens were examined at the Central Microbiology Laboratory of Hacettepe University. MALDI-TOF-MS (Biotyper IVD 4.2.80; Bruker Daltonics, GmbH, Germany) was used for species identification, and antibiotic susceptibility testing (AST) was performed by an automated method using the Phoenix TM M50 device (Becton Dickinson, Sparks, MD, USA). In the case of suspicious results, AST was repeated using disc diffusion, Mueller–Hinton agar or antibiotic gradient methods. All AST results were interpreted according to EUCAST criteria.^9^ There was a time lag of approximately 2–3 days between the MALDI-TOF result and the AST results.

### Statistical analysis

Descriptive statistics included median (IQR) for continuous variables and frequency (%) distributions for categorical variables. Coefficient of variation, kurtosis/skewness, histogram and Kolmogorov–Smirnov test were used to test the normality assumption. Comparisons were made using the Pearson’s chi-squared or the Fisher's exact tests. Mann–Whitney *U*-test was used to compare continuous variables between two groups. Effect modification was studied using the Breslow–Day test, and interaction terms in the logistic regression model. When building the models, any variable with a correlation greater than 40% was excluded from the model. Variables fulfilling criteria for potential confounders (age, time from hospital admission to specimen collection, invasive procedures) were adjusted for in logistic regression models. Effect sizes were presented as OR, with 95% CI.

Binary logistic regression models were constructed to identify predictors of ceftolozane/tazobactam resistance. Model 1 included age, time from hospital admission to specimen collection, history of chest tube, bronchoscopy, invasive mechanical ventilation and tracheostomy within the period from hospital admission to specimen collection, history of antibiotic use in the preceding month. Model 2 repeated Model 1, with addition of a new variable for preceding antibiotic use (restricted to antibiotics contributing significantly to the model). Model 3 was constructed to reach the highest prediction rates for ceftolozane/tazobactam resistance in *P. aeruginosa* isolated from respiratory specimens. This final model was used to calculate prediction scores, adopting a review of the current literature.^10–12^ The lowest beta coefficient in the final model was scored as 1; the beta coefficients of the other variables were divided by the lowest beta coefficient, and corresponding scores for these variables were obtained by rounding to the nearest whole number. Each study participant was given a ceftolozane/tazobactam resistance score, accordingly. The cut-off point of the score was determined as 2, using the Youden index. The sensitivity, specificity, positive predictive value (PPV) and negative predictive value (NPV) of the score were calculated according to this cut-off point. The performance of the score, Model 2 and Model 3 logistic regression predictive values were all assessed using ROC curves and AUC. Data were not imputed to replace missing data. A two-sided type 1 error level of 0.05 was used. Analyses were performed using the SPSS (IBM SPSS Corp, Armonk, NY, USA) version 29 package.

### Ethics

This study was approved by Hacettepe University Health Sciences Research Ethics Board (No: 2023/12-11). Informed consent was waived due to retrospective methodology, but all electronic health data were used anonymously at all stages of the study.

## Results

**Figure 1.**
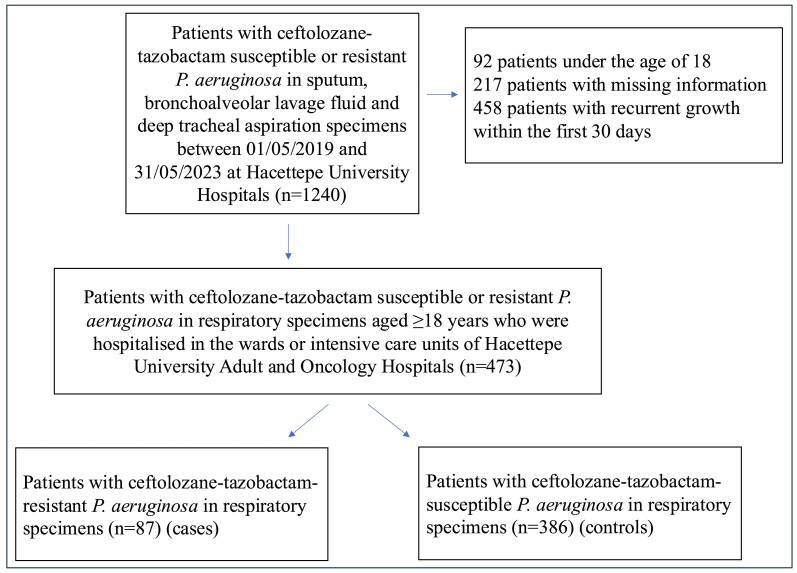
Flowchart of the study.

Of the 1240 patients in whom *P. aeruginosa* was isolated from sputum, BAL and/or DTA specimens, 473 patients met the inclusion criteria. Of these 473 patients, ceftolozane/tazobactam-resistant *P. aeruginosa* was detected in 87 patients (case group) and ceftolozane/tazobactam-sensitive *P. aeruginosa* was detected in 386 patients (control group) (Figure 1). *P. aeruginosa* isolates included in the study were obtained from DTA, sputum and BAL cultures, with percentages of 63.8%, 34% and 2.1%, respectively.

Of the 1240 patients, 37.6% were female. The median (IQR) age was 66 (18) years. The prevalence of ceftolozane/tazobactam resistance in *P. aeruginosa* isolates was 18.4%. At the time of sampling, 63.2% of patients had been receiving intensive care, 70.2% had received intensive care in the preceding 3 months and 55.8% had received invasive mechanical ventilation (Table 1).

**Table 1.** Demographic and clinical information of the patients included in the study

	*n* (%)	Median (IQR)
Age (years)		66 (56–74)
Female	178 (37.6)	
Charlson comorbidity index		3 (2–5)
Department in which the patient was hospitalized at the time of specimen collection		
Medical ward	135 (28.5)	
Surgical ward	32 (6.8)	
Emergency department	7 (1.5)	
ICU^a^	299 (63.2)	
Time from admission to specimen collection (days)		23 (7–49)
COVID-19 in the previous 3 months^b^	80 (21.6)	
Antibiotic use in the previous month	380 (80.3)	
Immunosuppressive treatment in the previous 3 months^c^	233 (49.3)	
Invasive procedures		
Bronchoscopy	52 (11.0)	
Chest tube insertion	55 (11.6)	
Tracheostomy	131 (27.7)	
Invasive mechanical ventilation	264 (55.8)	
Non-invasive mechanical ventilation	38 (8.0)	
Nasogastric catheterization^d^	274 (58.2)	
Gastrostomy tube insertion	89 (18.8)	
Central venous catheterization^e^	254 (54.0)	
Surgery in the previous 3 months	125 (26.4)	
Interventional radiological procedures in the previous 3 months	130 (27.5)	
Hospitalization in previous 3 months		
Ward	262 (55.4)	
ICU	332 (70.2)	
Albumin level (g/dL)		2.7 (2.4–3.1)
Transfer to ICU^f^	34 (19.9)	
Fourteen day fatality^g^	117 (26.1)	
Thirty day fatality^h^	184 (44.0)	

^a^Neurology, internal medicine, anaesthesia and surgical ICU.

^b^Three hundred and seventy-one patients with a sample date of 11 March 2020 and later were included.

^c^Steroids, chemotherapy or any other immunosuppressive agents.

^d^Information on nasogastric catheter use was not available for two patients.

^e^Information on central venous catheter use was not available for three patients.

^f^Three hundred and two patients who were in an ICU at the time of the specimen collection were excluded.

^g^Twenty-five individuals who were discharged in less than 14 days and for whom post-discharge survival information could not be obtained were not included in the analysis.

^h^Fifty-five people who were discharged in less than 30 days and for whom post-discharge survival information could not be obtained were not included in the analysis.

Distribution of demographic characteristics was similar between the cases and controls. Using internal medicine wards as a reference, the rate of ICU admission was higher among cases [OR (95% CI)= 2.0 (1.1–3.5), *P* = 0.045]. Admission to the oncology ICU was more frequent in cases, whereas the rate of admission to anaesthesia ICU was more frequent in controls (*P* < 0.001). The duration of hospitalization was longer in cases than in controls (*P* < 0.001). Cases were more likely to receive bronchoscopy [OR (95% CI) = 2.4 (1.3–4.6), *P* = 0.005], invasive mechanical ventilation [OR (95% CI) = 3.8 (2.2–6.5), *P* < 0.001], nasogastric catheters [OR (95% CI) = 2.6 (1.5–4.3), *P* < 0.001] and central venous catheters [OR (95% CI) = 3.5 (2.1–6.0), *P* < 0.001] compared with controls. A history of ward admission [OR (95% CI) = 1.8 (1.1–2.9), *P* = 0.047] and ICU admission in the preceding 3 months [OR (95% CI) = 3.5 (1.8–6.8), *P* = 0.047] was higher among cases than in controls (Table 2). MDR organisms (MDROs) were detected in 14.9% of cases, and XDR organisms (XDROs) or PDR organisms (PDROs) were reported for 79.3% of cases (*P* < 0.001).

**Table 2.** Demographic and clinical information of cases and controls

	Cases (*n* = 87)	Controls (*n* = 386)	*P* value	OR (95% CI)
Age (years), median (IQR)	64 (56–72)	67 (56–75)	0.189^a^	
Female, *n* (%)	34 (39.1)	144 (37.3)	0.758^b^	1.1 (0.7–1.7)
Charlson comorbidity index, median (IQR)	3 (2–5)	3 (2–4)	0.257^a^	
Department in which the patient was hospitalized at the time of specimen collection, *n* (%)			**0.045** ^c^	
Medical wards (reference)	17 (19.5)	118 (30.6)		1.0
Surgical wards	4 (4.6)	28 (7.3)		1.0 (0.3–3.2)
Emergency department	—	7 (1.8)		
ICU^d^	66 (75.9)	233 (60.4)		2.0 (1.1–3.5)
Time from admission to specimen collection (days), median (IQR)	34 (18–69)	19 (5–44)	**<0.001** ^a^	
Co-existence of Gram-negative microorganisms, *n* (%)	37 (42.5)	156 (40.4)	0.717^d^	1.1 (0.7–1.7)
COVID-19 in the previous 3 months, *n* (%)^e^	17 (23.3)	63 (21.1)	0.689^d^	1.1 (0.6–2.1)
Immunosuppressive treatment in the previous 3 months, *n* (%)^f^	51 (58.6)	182 (47.2)	0.053^d^	1.6 (1.0–2.5)
Invasive/non-invasive procedures, *n* (%)				
Bronchoscopy	17 (19.5)	35 (9.1)	**0.005** ^d^	2.4 (1.3–4.6)
Chest tube insertion	13 (14.9)	42 (10.9)	0.286^d^	1.4 (0.7–2.8)
Tracheostomy	30 (34.5)	101 (26.2)	0.117^d^	1.5 (0.9–2.4)
Invasive mechanical ventilation	69 (79.3)	195 (50.5)	**<0.001** ^d^	3.8 (2.2–6.5)
Non-invasive mechanical ventilation	3 (3.4)	35 (9.1)	0.082^d^	0.4 (0.1–1.2)
Nasogastric catheterization^g^	64 (75.3)	210 (54.4)	**<0.001** ^d^	2.6 (1.5–4.3)
Gastrostomy tube insertion	15 (17.2)	74 (19.2)	0.677^d^	0.9 (0.5–1.6)
Central venous catheterization^h^	67 (77.0)	187 (48.8)	**<0.001** ^d^	3.5 (2.1–6.0)
Surgery in the previous 3 months, *n* (%)	27 (31.0)	98 (25.4)	0.281^d^	1.3 (0.8–2.2)
Interventional radiological procedures in the previous 3 months, *n* (%)	28 (32.2)	102 (26.4)	0.277^d^	1.3 (0.8–2.2)
Hospitalization in the previous 3 months				
Ward, *n* (%)	58 (66.7)	204 (52.8)	**0.019** ^d^	1.8 (1.1–2.9)
Length of stay (days), median(IQR)^i^	17.5 (10–32)	16 (7–31)	0.318^a^	
ICU, *n* (%)	76 (87.4)	256 (66.3)	**<0.001** ^d^	3.5 (1.8–6.8)
Length of stay (days), median(IQR)^j^	28.5 (16–47)	22 (10–39)	**0.047** ^a^	
Albumin level (g/dL), median (IQR)	2.6 (2.4–2.9)	2.6 (2.4–3.1)	0.072^a^	
Fourteen day fatality, *n* (%)^k^	22 (25.6)	95 (26.2)	0.900^d^	1.0 (0.6–1.7)
Thirty day fatality, *n* (%)^l^	45 (54.2)	139 (41.5)	**0.037** ^d^	1.7 (1.1–2.7)

Bold type indicates statistical significance (*P* < 0.05).

^a^Mann–Whitney *U*-test.

^b^Pearson’s chi-squared test.

^c^Fisher’s exact test.

^d^Neurology, internal medicine, anaesthesia and surgical ICUs were analysed together.

^e^Three hundred and seventy-one patients with a sample date of 11 March 2020 and later were included.

^f^Steroids, chemotherapy or any other immunosuppressive agents.

^g^Information on nasogastric catheter use was not available for two patients.

^h^Information on central venous catheter use was not available for three patients.

^i^The analysis was carried out on 263 people who had been admitted to the ward in the previous 3 months; the hospitalization data of 10 patients could not be accessed because they had a history of hospitalization in an external centre.

^j^The analysis was performed on 331 individuals with a history of ICU hospitalization in the previous 3 months.

^k^Twenty-five individuals who were discharged in less than 14 days and for whom post-discharge survival information could not be obtained were not included in the analysis.

^l^Fifty-five people who were discharged in less than 30 days and for whom post-discharge survival information was not available were not included in the analysis.

A history of antibiotic consumption in the preceding month was detected in 94.3% of cases and 77.2% of controls [OR (95% CI) = 4.8 (1.9–12.3), *P* < 0.001]. The use of meropenem/imipenem, ceftazidime/avibactam, colistin/polymyxin B, tigecycline, amikacin/gentamicin, ciprofloxacin, levofloxacin, moxifloxacin and trimethoprim/sulfamethoxazole was significantly higher in the case group (Table 3).

**Table 3.** Antibiotic consumption in the previous month

	Cases *n* (%)	Controls *n* (%)	*P* value	OR (95% CI)
Antibiotic use in the previous month	82 (94.3)	298 (77.2)	**<0.001** ^a^	4.8 (1.9–12.3)
Carbapenems				
Meropenem/imipenem	66 (75.9)	164 (42.5)	**<0.001** ^a^	4.3 (2.5–7.2)
Ertapenem	4 (4.6)	7 (1.8)	0.125^b^	2.6 (0.7–9.1)
β-Lactam/β-lactamase inhibitors				
Ampicillin/sulbactam, amoxicillin/clavulanate	10 (11.5)	76 (19.7)	0.073^a^	0.5 (0.2–1.0)
Piperacillin/tazobactam	22 (25.3)	98 (25.4)	0.984^a^	0.9 (0.6–1.7)
Cefoperazone/sulbactam	3 (3.4)	12 (3.1)	0.745^b^	1.1 (0.3–4.0)
Ceftazidime/avibactam	4 (4.6)	2 (0.5)	**0.012** ^b^	9.3 (1.7–51.3)
Cephalosporins				
Ceftriaxone/cefixime	3 (3.4)	29 (7.5)	0.173^a^	0.4 (0.1–1.5)
Ceftazidime	4 (4.6)	15 (3.9)	0.763^b^	1.2 (0.4–3.7)
Cefepime	2 (2.3)	8 (2.1)	1.000^b^	1.1 (0.2–5.3)
Polymyxins				
Colistin/polymyxin	52 (59.8)	92 (23.8)	**<0.001** ^a^	4.7 (2.9–7.7)
Glycylcycline				
Tigecycline	16 (18.4)	29 (7.5)	**0.002** ^a^	2.8 (1.4–5.4)
Aminoglycosides				
Amikacin/gentamicin	16 (18.4)	32 (8.3)	**0.005** ^a^	2.5 (1.3–4.8)
Fluoroquinolones				
Levofloxacin/moxifloxacin/ciprofloxacin	16 (18.4)	34 (8.8)	**0.009** ^a^	2.3 (1.2–4.5)
Trimethoprim/sulfamethoxazole	11 (12.6)	16 (4.1)	**0.004** ^b^	3.3 (1.5–7.5)
Others^c^	3 (3.4)	16 (4.1)	1.000^b^	0.8 (0.2–2.9)

Bold type indicates statistical significance (*P* < 0.05).

^a^Pearson’s chi-squared test.

^b^Fisher’s exact test.

^c^Doxycycline, fosfomycin, cefuroxime.

Five of the seven patients considered as having a new episode were found to have received antibiotics for *P. aeruginosa* in the previous month. One of the seven patients had no history of antibiotic use and another patient had received antibiotherapy without antipseudomonal activity; these two patients were in the control group. The other patients with a history of antibiotic use in the previous month had been treated for infections caused by other organisms.

Two logistic regression models were constructed to evaluate factors associated with resistance to ceftolozane/tazobactam. Invasive mechanical ventilation [OR (95% CI) = 2.9 (1.6–5.4), *P* < 0.001] and bronchoscopy [OR (95% CI) = 2.1 (1.1–4.2), *P* = 0.026] were found to be associated with resistance to ceftolozane/tazobactam, after adjustment for age, time from admission to specimen collection, chest tube, tracheostomy and antibiotic use in the preceding month (Model 1, Table 4). When antibiotic use in the preceding month was included in Model 1, history of bronchoscopy [OR (95% CI) = 2.1 (1.1–4.3), *P* = 0.035], invasive mechanical ventilation [OR (95% CI) = 2.4 (1.2–4.5), *P* = 0.009], colistin/polymyxin B use in the preceding month [OR (95% CI) = 3.2 (1.8–5.7), *P* < 0.001] and fluoroquinolone use in the preceding month [OR (95% CI) = 2.3 (1.1–4.8), *P* = 0.024] were significantly associated with resistance to ceftolozane/tazobactam (Model 2, full model, Table 4).

**Table 4.** Risk factors associated with ceftolozane/tazobactam resistance in *P. aeruginosa* strains isolated from respiratory specimens; results of multivariate analysis (binary logistic regression)

	Model 1				Model 2 (full model)		
	OR	95% CI	*P* value		OR	95% CI	*P* value
Age (years)	1.0	1.0–1.0	0.389	Age (years)	0.9	0.9–1.0	0.561
Time from admission to sample collection (days)	1.0	1.0–1.0	0.022	Time from admission to specimen collection (days)	1.0	1.0–1.1	0.054
Tracheostomy	0.7	0.4–1.3	0.293	Tracheostomy	0.6	0.3–1.1	0.115
Chest tube insertion	1.0	0.5–2.1	0.976	Chest tube insertion	1.1	0.5–2.2	0.895
Bronchoscopy	2.1	1.1–4.2	**0.026**	Bronchoscopy	2.1	1.1–4.3	**0.035**
Invasive mechanical ventilation	2.9	1.6–5.4	**<0.001**	Invasive mechanical ventilation	2.4	1.2–4.5	**0.009**
Antibiotic use in the previous month	2.5	0.9–6.6	0.072	Colistin/polymyxin B use in previous month	3.2	1.8–5.7	**<0.001**
				Fluoroquinolone use in the previous month	2.3	1.1–4.8	**0.024**

Bold type indicates statistical significance (*P* < 0.05). Number of cases for each model:87; total number:473. Model 1; Hosmer–Lemeshow test *P* value = 0.762, Nagelkerke R^2^ = 0.140, −2 log likelihood = 408.802. Model 2; Hosmer–Lemeshow test *P* value = 0.304, Nagelkerke R^2^ = 0.201, −2 log likelihood = 389.068.

**Figure 2.**
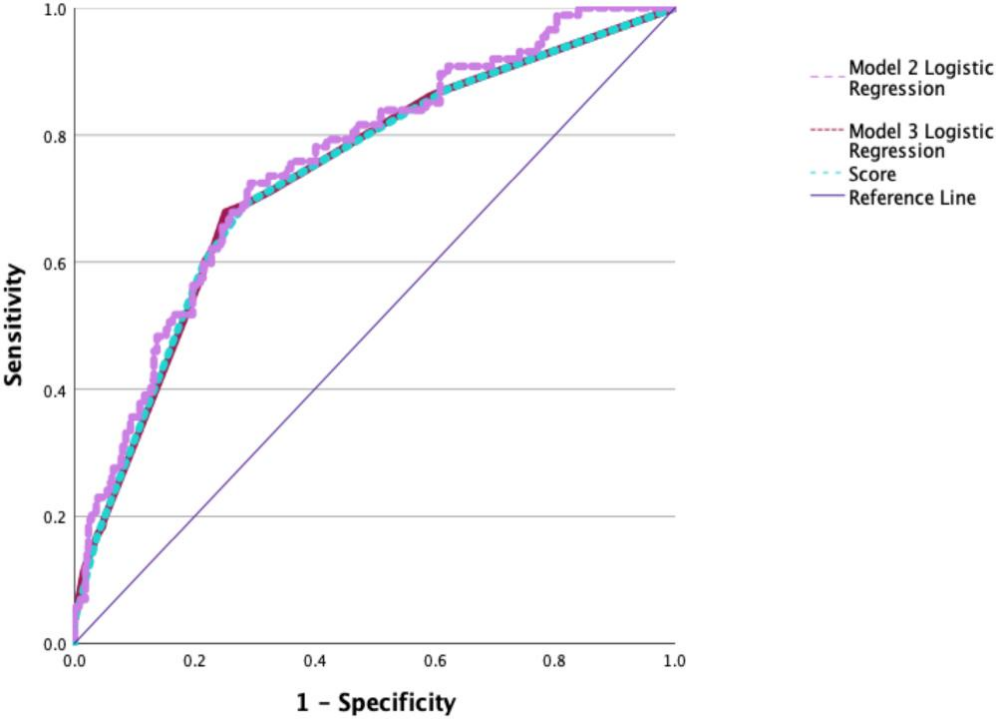
Comparison of ROC curves of Model 2, Model 3 and the scoring system for predicting ceftolozane/tazobactam resistance in patients with *P. aeruginosa* in respiratory specimens. Model 2 (logistic regression): AUC (95% CI) = 0.754 (0.698–0.810), *P* < 0.001; PPPV = 60.9%; NPV = 83.8%; sensitivity = 16.1%; specificity = 97.7%. Model 3 (logistic regression): AUC (95% CI) = 0.735 (0.675–0.795), *P* < 0.001; PPV = 50%; NPV = 83.7%; sensitivity = 17.2%; specificity = 96.1%. Score: AUC (95% CI) = 0.734 (0.675–0.794), *P* < 0.001; PPV = 35.5%; NPV = 91.1%; sensitivity = 69.0%; specificity = 71.8%.

According to Model 3 (final model) constructed in terms of prediction (score calculation), history of bronchoscopy was scored as 1, history of invasive mechanical ventilation as 1, fluoroquinolone use in the preceding month as 1 and colistin use in the preceding month as 2 points. The score ranged from 0 to 5 (Table 5). The AUC (95% CI) of the ROC curves obtained from Model 2 and Model 3 and the final score were similar (Figure 2). The Youden index cut-off was set at 2, i.e. a score of ≥2 indicates resistance to ceftolozane/tazobactam and a score of <2 indicates susceptibility to ceftolozane/tazobactam. The AUC (95% CI) of the score was 0.734 (0.675–0.794), with a sensitivity of 69%, specificity of 71.8%, PPV of 35.5% and NPV of 91.1%.

**Table 5.** Model 3 (final model) and scores for prediction of ceftolozane/tazobactam resistance in *P. aeruginosa* strains isolated from respiratory specimens

	Model 3				Score calculation	Score
	Beta	OR	95% CI	*P* value		
Bronchoscopy	0.766	2.15	1.08–4.29	0.029	1	1
Invasive mechanical ventilation	0.807	2.24	1.19–4.21	0.012	0.807/0.766 = 1.1	1
Colistin/polymyxin B use in previous month	1.224	3.40	1.96–5.89	<0.001	1.224/0.766 = 1.6	2
Fluoroquinolone use in the previous month	0.839	2.31	1.13–4.73	0.022	0.839/0.766 = 1.1	1

Model 3; Hosmer–Lemeshow test *P* value = 0.924, Nagelkerke R^2^ = 0.186, −2 log likelihood = 394.126.

### Subgroup analyses

*P. aeruginosa* was identified as the single pathogen in 53.3% of respiratory specimens, while polymicrobial growth was detected in 46.7% of the respiratory specimens. In a subgroup analysis restricted to monomicrobial-positive specimens (*n* = 252), there were no statistically significant differences in distributions of age, sex or comorbidity across the groups. Time from admission to specimen collection (*P* < 0.001), use of invasive mechanical ventilation [OR (95% CI) = 3.4 (1.7–7.0), *P* < 0.001], nasogastric catheter [OR (95% CI) = 2.4 (1.2–4.8), *P* = 0.008] and central venous catheter [OR (95% CI) = 3.4 (1. 7–6.8), *P* < 0. 001], a history of a ward stay [OR (95% CI) = 2.3 (1.2–4.6), *P* = 0.013] or an ICU stay [OR (95% CI) = 3.2 (1.4–7.2), *P* = 0.004] of more than 72 h in the preceding 3 months were all significantly higher in cases. Among patients with monomicrobial growth, 91.5% of cases and 74.1% of controls had a history of antibiotic use in the preceding month [OR 95% CI = 3.7(1.3–10.9), *P* = 0.010]. The use of meropenem/imipenem, colistin/polymyxin B and fluoroquinolones was significantly more frequent in the case group.

In multivariate analysis, colistin/polymyxin B use [OR (95% CI) = 3.1 (1.3–7.5) *P* = 0.012] and meropenem/imipenem use in the preceding month [OR (95% CI) = 2.8 (1.1–7.0), *P* = 0.027] were both associated with ceftolozane/tazobactam resistance, whereas tracheostomy was protective against ceftolozane/tazobactam resistance [OR (95% CI) = 0.2 (0.1–0.7), *P* = 0.005], adjusting for age, time from admission to specimen collection, and use of chest tube, bronchoscopy and invasive mechanical ventilation.

## Discussion

In this study, a history of bronchoscopy, invasive mechanical ventilation, colistin/polymyxin B or fluoroquinolone use in the preceding month were identified as factors associated with ceftolozane/tazobactam resistance in the multivariate analysis. The predictive scoring system proposed was shown to have a sensitivity of 69% and specificity of 71.8%, with PPV and NPV of 35.5% and 91.1%, respectively. Although the PPV is low, considering that predictive values vary according to prevalence, the PPV will be higher in areas where resistance to ceftolozane/tazobactam is higher. Ceftolozane/tazobactam was not in use in our country during the data collection period; it is currently approved for use but not reimbursed. In our study, resistance to ceftolozane/tazobactam was 18.4% in a region where ceftolozane/tazobactam was not used. Considering that the prevalence of resistance will change with increasing use, a better approach is to evaluate the performance of scoring systems on the basis of sensitivity and specificity.

Primary research on risk factors for ceftolozane/tazobactam resistance is scarce. Tamma *et al.*^6^ investigated modifiable risk factors for ceftolozane/tazobactam resistance in patients receiving ceftolozane/tazobactam for carbapenem-resistant *P. aeruginosa* infections. Inadequate source control and ceftolozane/tazobactam infusion of less than 3 h were reported as modifiable risk factors for resistance to ceftolozane/tazobactam. Interestingly, 86% of index isolates susceptible to ceftazidime/avibactam subsequently developed resistance to it after exposure to ceftolozane/tazobactam. The development of resistance to ceftazidime/avibactam without exposure to ceftazidime/avibactam suggests that exposure to a broad-spectrum antibiotic may induce resistance to antibiotics to which the bacteria have never been exposed. Similarly, in our study, exposure to fluoroquinolones and polymyxins was associated with ceftolozane/tazobactam resistance in our patients who had never been exposed to ceftolozane/tazobactam. In our univariate analyses, a history of ceftazidime/avibactam use was higher in patients with ceftolozane/tazobactam resistance, but this association became non-significant after adjustment for other factors. This could also be a type 2 error due to the limited number of patients with a history of ceftazidime/avibactam use. Although ceftazidime/avibactam was in clinical use during the data collection period, it was only used in ICUs, with limited indications.

In a study by Meschiari *et al.*,^7^ age, previous *P. aeruginosa* colonization, length of hospital stay, urinary tract infection and previous exposure to carbapenems were found to be independent risk factors associated with resistance to both ceftolozane/tazobactam and ceftazidime/avibactam.^7^ This study has similarities and differences with our study. In our study, imipenem/meropenem use in the preceding month was associated with ceftolozane/tazobactam resistance in both univariate and multivariate analyses, in monomicrobial isolates. In analyses including polymicrobial isolates, carbapenem use was associated with ceftolozane/tazobactam resistance in univariate analyses, but this association was not maintained in multivariate analyses. In patients with polymicrobial growth, carbapenems might have been used more intensively for both cases and controls, diminishing the differences in use between groups (with a potential bias towards the null).

In a setting where resistance to ceftolozane/tazobactam is widespread and compliance with infection control measures is low, resistant *P. aeruginosa* colonization is expected to increase with prolonged hospital stay. Although duration of hospitalization before specimen collection was associated with ceftolozane/tazobactam resistance in our univariate analyses, it lost significance in multivariate analyses. This difference was thought to be due to differences in local resistance data or adherence to infection control measures.

Our proposed scoring method could be useful for predicting resistance in patients with *P. aeruginosa* infection. High NPVs may allow clinicians to initiate ceftolozane/tazobactam appropriately while awaiting AST results. In a systematic review evaluating clinical prediction scores, models exhibiting sensitivity, specificity, PPV and NPV exceeding 75%, an AUC value exceeding 0.7 and a Hosmer–Lemeshow *P* value exceeding 0.3 were considered to demonstrate optimal model performance.^13^ In accordance with the above criteria, the logistic model (Model 3) and ROC curves used for prediction showed that the performance of the scoring system was satisfactory, except for the PPV. This may be related to the relatively low incidence of ceftolozane/tazobactam resistance associated with the absence of ceftolozane/tazobactam use in the hospital and in the country during the study period.

Subgroup analyses revealed similar predictors of ceftolozane/tazobactam resistance in the presence of monomicrobial or polymicrobial growth. The subgroup analyses indicated that tracheostomy may confer a protective effect against resistance to ceftolozane/tazobactam, which was in line with the findings of previous studies that suggested that early tracheostomy may prevent ventilator-associated pneumonia (VAP).^14,15^ It is anticipated that a decline in the prevalence of VAP will result in a reduction in the overall consumption of antibiotics and, consequently, contribute to a decrease in antibiotic resistance.

A limitation of this study is that data were collected retrospectively from hospital electronic health records. Despite all potential data linkages across patients’ medical history, consultation notes and laboratory records, (missing) information bias cannot be thoroughly excluded.

Clonal spread of *P. aeruginosa* and resistance mechanisms, which have the potential to explain cross-resistance, were not investigated.

Inadequate disinfection, a deviation from the original disinfection protocol, and damage in bronchoscope channels have been identified as potential contributing factors in the onset *of P. aeruginosa* outbreaks.^16^ The impact of these variables could not be investigated due to the retrospective methodology of current the study.

This study was conducted as a single centre in a tertiary care referral centre. Therefore, the findings cannot be generalized to all hospitals. Patients under 18 years of age and outpatients were not included in the study, thus the results cannot be extended to these groups.

This study is notable for its contribution to the limited body of research investigating the risk factors associated with resistance to ceftolozane/tazobactam. The study offers valuable insights into the prevalence of ceftolozane/tazobactam resistance in a tertiary care hospital, particularly in a period preceding the introduction of ceftolozane/tazobactam in our country. All respiratory specimens were tested in the same microbiology laboratory using the same methodology and in accordance with the same guidelines, thus ensuring comparability of cases and controls.

In conclusion, to the best of our knowledge this is the first study that has investigated a prediction score to examine ceftolozane/tazobactam resistance in respiratory isolates of *P. aeruginosa*. The score can be easily implemented at the bedside. It may enable clinicians to refrain from initiating unnecessary ceftolozane/tazobactam treatment until the AST results become available. The evaluation of the performance of the score based on sensitivity, specificity and the area under the ROC curve indicates that this approach can be effective in settings where the rate of ceftolozane/tazobactam resistance is higher than in our hospital. Although the PPV of the score was found to be low, the PPV is expected to increase in regions with high prevalence of resistance to ceftolozane/tazobactam.

## Data Availability

The data underlying this article will be shared on reasonable request to corresponding author.
